# New Remedy

**Published:** 1831

**Authors:** 


10. *New Remedy.*—M. Jumeret Perrault has announced the discovery of a plant in the mountains of Neufchatel, which is a sovereign remedy for pulmonary complaints. He offers it at seventeen pence per package, and supposes it to be a species of *asplenium.*—*Ib.*

